# Loss of transcription factor *Zbtb33* fails to induce clonal hematopoiesis but plays a role in tumor growth

**DOI:** 10.1242/dmm.052484

**Published:** 2026-10-26

**Authors:** Yuhang Li, Kaiping Luo, Keyao Xia, Jingjing Liu, Zhigang Zhao, Zhigang Cai, Ge Dong

**Affiliations:** ^1^ State Key Laboratory of Experimental Hematology, Tianjin Medical University, Tianjin 300037, China; ^2^ Tianjin Key Laboratory of Inflammatory Biology, Department of Pharmacology, School of Basic Medical Science, Tianjin Medical University, Tianjin 300037, China; ^3^ The Province and Ministry Co-sponsored Collaborative Innovation Center for Medical Epigenetics, School of Basic Medical Science, Tianjin Medical University, Tianjin 300037, China; ^4^ Department of Medical Oncology, Tianjin First Central Hospital, Affiliated Hospital of Nankai University, Tianjin 300192, China; ^5^ Department of Medical Oncology, Tianjin First Central Hospital, School of Medicine, Nankai University, Tianjin 300037, China

**Keywords:** Clonal hematopoiesis, *Zbtb33*, *Tet2*, *P53*, Inflammation, Tumor growth

## Abstract

Clonal hematopoiesis (CH) is an early indicator of hematologic malignancies, driven by mutations in hematopoietic stem cells (HSCs), such as those in *TET2 or P53*. Recently, *ZBTB33* mutations have been implicated in myelodysplastic syndromes and suggested as a driver of CH. However, the role of *ZBTB33* in hematopoiesis and its involvement in CH remains unclear. We generated a *Zbtb33*-knockout mouse strain to elucidate the function of *Zbtb33* in hematopoiesis and tumor growth. Our findings indicated that hematopoiesis in *Zbtb33*-knockout mice was normal, and competitive bone marrow transplantation assays demonstrated that loss of *Zbtb33* in HSCs did not confer expansional advantage. Introducing *Zbtb33* mutation into *Tet2*- or *P53*-mutation background yielded no synergistic effects. Nevertheless, tumor-bearing experiments revealed that hematopoietic loss of *Zbtb33* accelerated solid tumor growth. In summary, *Zbtb33* knockout was insufficient to induce CH in mice but may play a role in tumor growth through the hematopoietic compartment.

## Teaser

**Summary:** In mice, *Zbtb33* plays a limited role in hematopoiesis, but its involvement in tumor immunity requires further investigation.

## INTRODUCTION

Clonal hematopoiesis (CH) is an age-associated aberrant hematopoiesis phenomenon characterized by the presence of certain somatic mutations in hematopoietic stem cells (HSCs), leading to the clonal expansion advantages of these cells and an increase in the proportion of mutant blood cells in the circulation (Jaiswal and Libby, 2020; von Bonin et al., 2021). CH is recognized as a pivotal precursor to various hematological malignancies, such as myelodysplastic syndromes (MDS) and acute myeloid leukemia (AML), as well as an independent risk factor for several non-hematological chronic diseases, such as cardiovascular diseases, colitis and autoimmunity diseases (typically, autoinflammatory diseases such as VEXAS syndrome) (Dong et al., 2025; Molteni et al., 2025). Early detection of CH could potentially allow for interventions that prevent or delay the onset of associated diseases (Stengel et al., 2021; Beauchamp et al., 2021). Clonal hematopoiesis of indeterminate potential (CHIP) is a concept defined by the presence of specific somatic mutations in peripheral blood (PB) with variant allele fractions (VAFs) exceeding 2% (Beauchamp et al., 2021; Jaiswal et al., 2014). Genes such as *TET2*, *DNMT3A*, *P53* (also known as *TP53*) and *JAK2* are among the well-known drivers of CH, and have been identified in recent clinical and epidemiological studies (Beeler et al., 2026; Gerhardt et al., 2026; Mon Père et al., 2026; Baeten et al., 2026), while *UBA1^M41L^* is a recently identified new driver of CH (Dong et al., 2025; Molteni et al., 2025). Importantly, bona fide CH drivers with validations from experimental settings using mouse models and/or human cell models support their causal role in the pre-cancer/pre-leukemic state (Evans and Walsh, 2023; Jakobsen et al., 2024).

Zinc finger and BTB domain containing 33 (ZBTB33; also known as KAISO) is a member of the BTB/POZ subfamily of zinc finger transcription factors. Aberrant ZBTB33/KAISO expression has been observed in multiple hematopoietic malignancies, such as chronic myeloid leukemia, chronic myelomonocytic leukemia-like and atypical chronic myelomonocytic-like at the molecular level (Palomo et al., 2020). A recent study has also suggested that *ZBTB33* is linked to poor outcomes in acute T lymphocytic leukemia (García-Hernández et al., 2023). Additionally, *ZBTB33* has been implicated in a variety of human malignancies beyond hematopoietic systems, including prostate (Jones et al., 2012; Wang et al., 2016), breast (Bassey-Archibong et al., 2017; Jones et al., 2014), pancreatic (Jones et al., 2016) and lung (Dai et al., 2009) cancers. Its involvement in these cancers indicates a broad impact of *ZBTB33* on tumorigenesis.

The origin (i.e. drivers) and dynamics (i.e. history, trajectory and outcomes) of CH are central to understanding its role in the development of hematological malignancies and other diseases (Niroula et al., 2021; Jaiswal and Ebert, 2019; Bowman et al., 2018). Our previous studies, corroborated by recent related studies, have suggested that *Tet2* mutations in HSCs lead to three distinct outcomes: (1) increased self-renewal capability; (2) myeloid-skewing differentiation; (3) increased inflammation (Wen et al., 2024). In our most recent study, we demonstrated that dextran sulfate sodium (DSS)-induced colitis drives *Tet2*-deficiency-mediated CH in a *Tet2*-mutant background (He et al., 2025). In the present study, we investigated whether the combination of *Zbtb33* mutation and *Tet2* mutation promotes CH and leukemogenesis.

A study by Beauchamp et al. (2021) showed *ZBTB33* to be a driver of CH in human, using a tailored pipeline to unbiasedly integrate diverse human PB DNA-sequencing datasets from the Exome Aggregation Consortium (ExAC) and Trans-Omics for Precision Medicine (TOPMed) (Beauchamp et al., 2021). Bernstein et al. (2024) later reported that *ZBTB33* is one of 17 drivers of CH in human, based on clinical epidemiology rather than experimental validations (Bernstein et al., 2024). Moreover, a mutation pattern in *ZBTB33* has been recurrently detected with high VAFs in multiple clinical trials of myeloproliferative neoplasms (MPNs)/MDS (Stengel et al., 2021; Beauchamp et al., 2021), which are inclined towards malignant transformation (i.e. AML) (Nangalia et al., 2013; Tyner et al., 2018). These studies suggest a potential role of *ZBTB33* in CH and CH-related hematopoietic malignancies. However, it is important to note that these studies establish ‘association’ rather than ‘causality’. Thus, the question of whether HSCs carrying *ZBTB33* loss-of-function (LOF) mutations enhance self-renewal and expansion advantages remains to be clarified. Furthermore, there is a need for systematic and *in vivo* investigation into the role of *ZBTB33* in hematopoiesis and in other biological processes.

Here, we generated *Zbtb33*-knockout (KO) mice to explore the potential alterations in hematopoiesis under physiological conditions or in response to external stimuli. Our experimental results do not support the hypothesis that *Zbtb33* mutations act as drivers of CH; moreover, our results suggest that loss of *Zbtb33* does not exhibit synergistic effects with the loss of *Tet2* or *P53* (also known as *Trp53* in mouse). Our preliminary data suggest that *Zbtb33* has equally abundant expression in myeloid and T cells, and loss of *Zbtb33* in the tumor microenvironment resulted in mitigated tumor growth, suggesting a role for *Zbtb33* in anti-tumor growth.

## RESULTS

### Generation of *Zbtb33*-KO mice and monitoring of the PB of male mutants (*Zbtb33^−/y^*)

Using CRISPR/Cas9 technology, two sgRNAs were designed to target the core region of *Zbtb33* exon 2 in which protein-coding sequences initiate (note that *Zbtb33/ZBTB33* is located on X chromosome in mouse and human; Fig. S1A). Recognizing that somatic mutations in *ZBTB33* (LOF) are predominantly identified in male donors with CH (Bernstein et al., 2024), this study focused on male mutants (*Zbtb33*^−*/y*^). High-quality antibodies for ZBTB33 are unavailable; therefore, we confirmed transcriptional depletion of *Zbtb33* by bulk RNA sequencing (RNA-seq) of wild-type (WT) and mutant bone marrow cells (BMCs) (Fig. 1A).

**Fig. 1.**
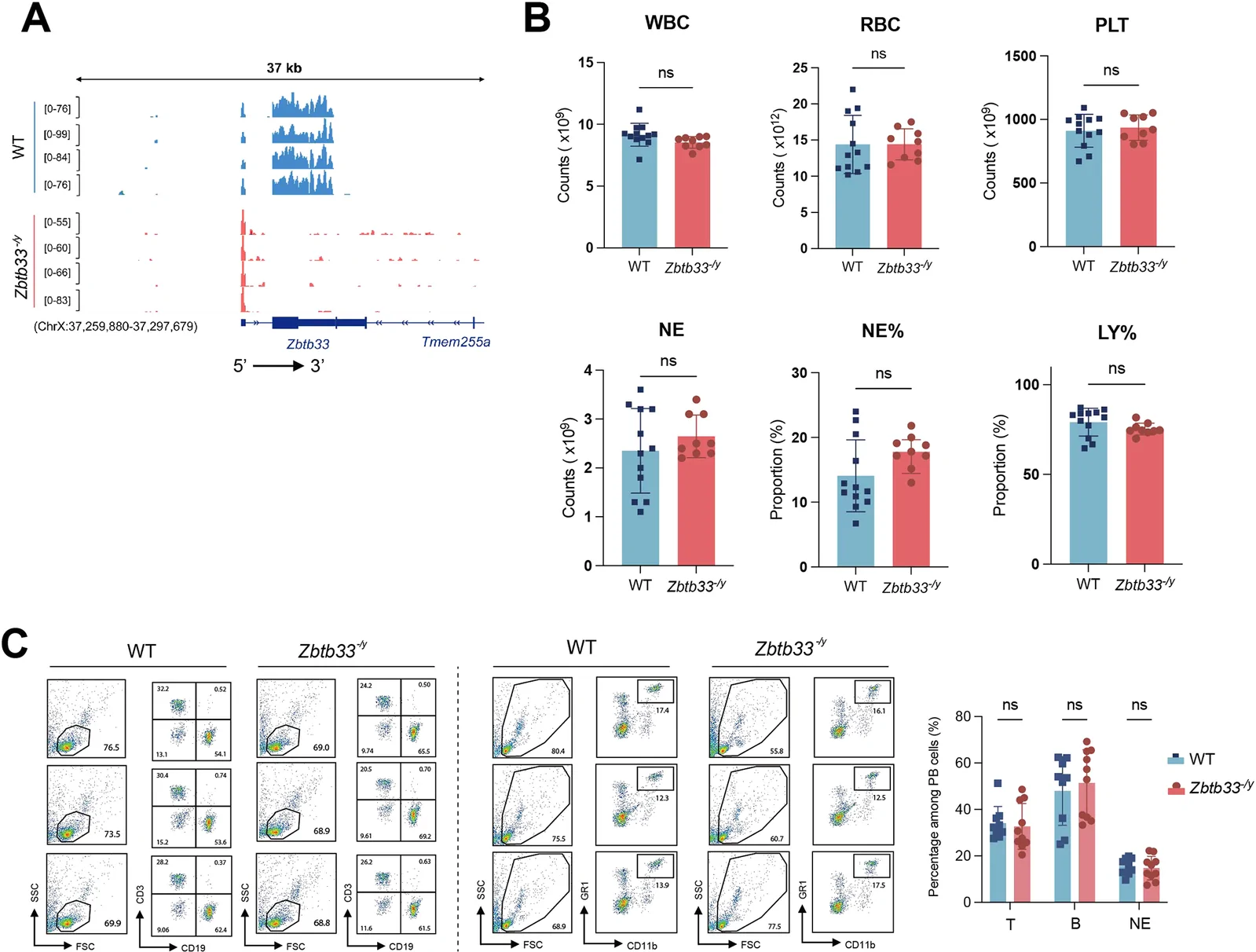
**Generation of *Zbtb33* knockout mice and monitoring of *Zbtb33^−/y^* peripheral blood.** (A) Read alignment using bulk RNA-sequencing datasets of bone marrow cells (BMCs) from *Zbtb33*-deficient and wild-type (WT) mice. Region for *Zbtb33* locus (exons 1 and 2) is shown. Exon 1 is preserved, but exon 2 is absent, in *Zbtb33^−/y^* (mutant) mice, which is consistent with the targeting strategy (see Fig. S1A). (B) Peripheral blood (PB) cell counts for adult *Zbtb33*-deficient mice and WT littermates. LY%, lymphocyte proportion; NE, absolute neutrophil count; NE%, neutrophil proportion; PLT, platelets; RBC, red blood cells; WBC, white blood cells. (C) Representative flow cytometry profiles and quantification of T cells (T), B cells (B) and neutrophils (NE) in PB. Cell counts and flow cytometry analysis suggest that *Zbtb33*^−/y^ mice have normal blood cell compositions. FSC, forward scatter; SSC, side scatter. ns, not significant (unpaired, two-tailed Student's *t*-test). WT, *n*=12; *Zbtb33^−/y^*, *n*=9; *n* denotes the number of biological replicates.

We performed a comprehensive phenotypic assessment of *Zbtb33*^−*/y*^ male mice. PB parameters were monitored throughout their lifetime and appeared normal compared to those of controls. At 6-8 months of age, hematological parameters [including white blood cells (WBCs), red blood cells, platelets, absolute neutrophil count (NE), neutrophil proportion (NE%) and lymphocyte proportion (LY%)] showed no significant differences between mutant and WT mice (Fig. 1B). These findings are in concordance with previous findings reported by an independent research group (Prokhortchouk et al., 2006). Flow cytometry analysis also revealed no significant abnormalities in proportions of T cells (CD19^−^; CD3^+^), B cells (CD19^+^; CD3^−^) or neutrophils [CD11b^+^ (also known as ITGAM^+^); GR1^+^ (also known as GSR1+)] in the PB of *Zbtb33^−/y^* mice (Fig. 1C). These findings were consistent across extended ages (6-12 months), in which we confirmed that these parameters remained within the normal ranges (Fig. S1B). In summary, *Zbtb33*-deficient male mice exhibited no marked alterations in PB parameters throughout the examined age range (2-12 months). Moreover, complete blood count analyses in *Zbtb33^+/−^*, *Zbtb33^−/−^* and WT female mice revealed no significant differences among the three genotypes, indicating that *Zbtb33* haploinsufficiency does not overtly disrupt steady-state peripheral hematopoiesis (Fig. S1C).

### Loss of *Zbtb33* is insufficient to affect HSCs in bone marrow

To investigate the impact of *Zbtb33* deficiency on developmental hierarchy and cellularity of bone marrow, we compared the proportions of hematopoietic stem and progenitor cell (HSPC) subsets between *Zbtb33*-KO mice and WT littermates using cytometric analyses (Fig. 2A-F). No statistically significant differences were observed in the proportions of HSPC subtypes, although a slight, non-significant increase in the proportion of Lin^−^ cells was noted in *Zbtb33*-KO mice (Fig. 2A, *P*=0.09; Fig. S1D). Similarly, the proportions of Lin^−^Sca1^+^Kit^+^ (LSK) cells and their subpopulations, including HSCs (LSK-CD150^+^CD48^−^), remained comparable between groups (Fig. 2B-G; Fig. S1D). Given a potential role for ZBTB33 in myeloid hematological disorders, such as MDS/MPNs, we further examined myeloid hematopoietic progenitor subpopulations. Again, no significant differences were found in the proportions of myeloid progenitors (MPs; Lin^−^Sca1^−^cKit^+^) and common myeloid progenitors (CMPs; Lin^−^Sca1^−^cKit^+^CD16^−^CD34^+^) between *Zbtb33*-KO mice and WT mice (Fig. 2H; Fig. S1E). However, in terms of the mature cells in bone marrow, the proportion of neutrophils was significantly higher in *Zbtb33*-KO mice than in WT mice, whereas T cells (CD3^+^) and B cells (CD19^+^) remained unchanged, suggesting selective enhancement of neutrophil differentiation (Fig. 2I, *P*<0.05). Collectively, these findings suggested that the loss of *Zbtb33* was insufficient to affect the proliferation and expansion of HSPCs but moderately enhanced cell differentiation and maturation of certain myeloid lineages (neutrophils) in bone marrow.

**Fig. 2.**
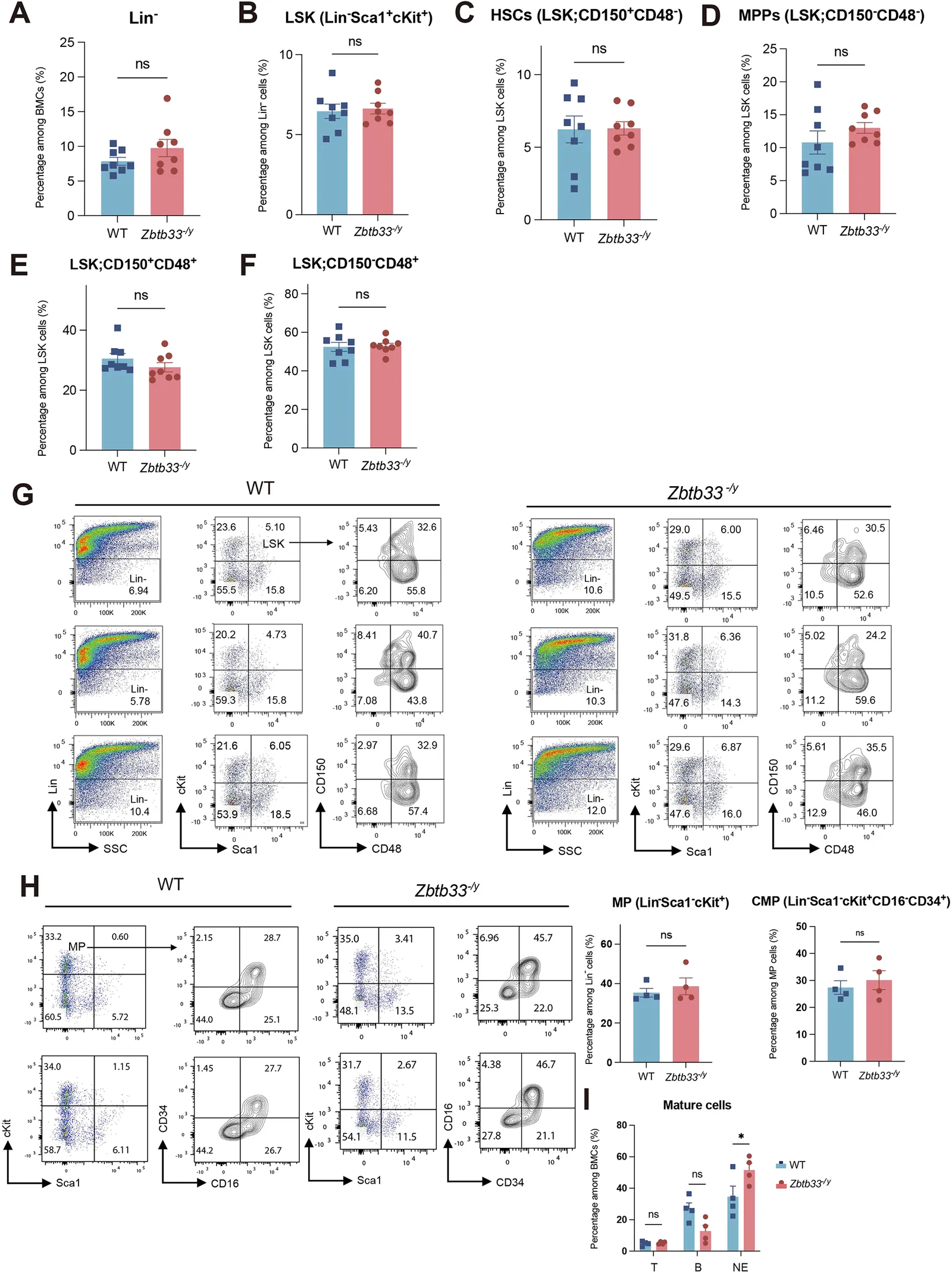
**Loss of *Zbtb33* subtly affects hematopoietic stem and progenitor cells in bone marrow.** (A) Percentage of Lin^−^ cells in the bone marrow of *Zbtb33*-knockout (KO) mice compared with that in the bone marrow of WT littermates. WT, *n*=8; *Zbtb33^−/y^*, *n*=8; *n* denotes the number of biological replicates. (B) Percentage of Lin^−^Sca1^+^cKit^+^ (LSK) cells in the Lin^−^ cells in the BM. WT, *n*=8; *Zbtb33^−/y^*, *n*=8; *n* denotes the number of biological replicates. (C-F) Percentages of hematopoietic stem cells (HSCs; Lin^−^Sca1^+^cKit^+^CD150^+^CD48^−^) (C), multipotent progenitors (MPPs; Lin^−^Sca1^+^cKit^+^CD150^−^CD48^−^) (D), Lin^−^Sca1^+^cKit^+^ CD150^+^CD48^+^ cells (E) and Lin^−^Sca1^+^cKit^+^ CD150^−^CD48^+^ cells (F) in *Zbtb33*-KO mice compared with those in WT littermates. WT, *n*=8; *Zbtb33^−/y^*, *n*=8; *n* denotes the number of biological replicates. (G) Representative flow cytometry profiles of hematopoietic stem and progenitor cells (HSPCs) in bone marrow. (H) Representative flow cytometry profiles and quantification of myeloid progenitors (MP) and common myeloid progenitors (CMP) in bone marrow. WT, *n*=4; *Zbtb33^−/y^*, *n*=4; *n* denotes the number of biological replicates. (I) Flow cytometry quantification of T cells, B cells and neutrophils in bone marrow. ns, not significant; *P<0.05 (unpaired, two-tailed Student's *t*-test).

In older *Zbtb33*-KO mice (6-12 months), most HSPC subsets and myeloid progenitors showed no statistically significant differences from those in aged WT controls (Fig. S1F,G). However, among the HSPC subpopulations, the Lin^−^Sca1^+^cKit^+^CD150^+^CD48^+^ population was significantly reduced in the bone marrow of aged *Zbtb33*-KO mice compared to that in aged WT mice (Fig. S1F, *P*=0.0218). Mature cell populations in the bone marrow remained comparable between aged *Zbtb33*-KO and WT mice (Fig. S1H). Overall, *Zbtb33* deficiency exerted negligible impact on the expansion of HSPCs and myeloid progenitors in bone marrow, regardless of age.

### *Zbtb33* mutant HSCs have no competitive advantage in competitive transplantation experiments

To assess the hematopoietic reconstitution and expansion potential of *Zbtb33*-deficient HSPCs, competitive bone marrow transplantation (cBMT) assays were performed using a mixture of *Zbtb33* mutant and WT HSCs in recipients. BMCs from *Zbtb33-*KO or WT donor mice (CD45.2^+^) were mixed with competitor BMCs from Boy/J mice (CD45.1^+^) at either a 1:1 or 1:5 donor-to-competitor ratio and transplanted into lethally irradiated recipient mice (CD45.1^+^CD45.2^+^). All recipient mice successfully reconstituted hematopoiesis and survived throughout the observation period, indicating that loss of *Zbtb33* does not impair hematopoietic reconstitution capacity. PB chimerism was monitored by flow cytometry to quantify CD45.2^+^ donor-derived cells (Fig. 3A). Under the 1:1 competitive condition, *Zbtb33*-KO donor cells exhibited consistently lower PB chimerism than did WT donor cells during long-term follow-up, suggesting that *Zbtb33* deficiency does not confer a clonal expansion advantage to HSCs (Fig. 3B,C). Secondary transplantation further amplified this difference, with *Zbtb33*-KO donor cells displaying even greater reduction in chimerism relative to that in WT controls (Fig. 3C). Consistent results were observed under the 1:5 competitive condition, in which *Zbtb33*-KO donor cells again demonstrated significantly reduced chimerism compared to that in WT counterparts, regardless of the donor-to-competitor ratio (Fig. 3E). Moreover, at the endpoint, significant reductions in neutrophils and B cells of PB were observed in recipients transplanted with *Zbtb33*-deficient BMCs (Fig. 3D). Further analysis showed no lineage bias, with all PB cell types exhibiting low chimerism (Fig. 3F-H).

**Fig. 3.**
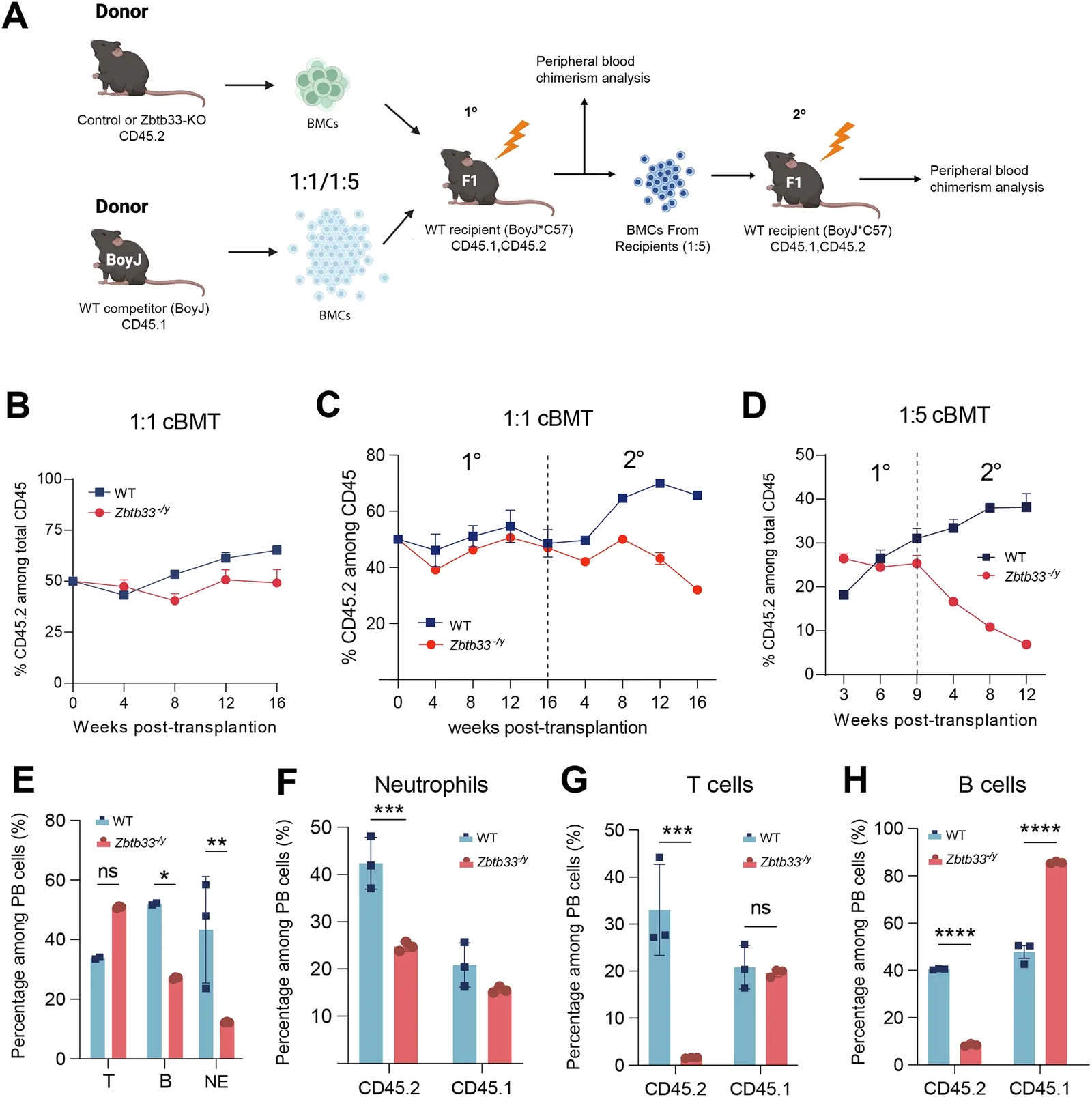
***Zbtb33* mutant HSCs have no competitive advantage in competitive bone marrow transplantation assays.** (A) Schematic diagram of competitive bone marrow transplantation (cBMT) assay with a 1:1 or 1:5 ratio. (B) PB chimerism at the indicated time points with a 1:1 BMC mixing ratio. (C) PB chimerism at the indicated time points post cBMT with a 1:1 BMC mixing ratio. A secondary cBMT was performed, with the primary and secondary cBMT separated by the dashed line. (D) PB chimerism at the indicated time points post cBMT with a 1:5 BMC mixing ratio. A secondary cBMT was performed, with the primary and secondary cBMT separated by the dashed line. (E) Frequencies of the indicated cell types in PB at 12 weeks post cBMT. (F-H) Flow cytometry quantification of the lineage chimerism analysis of CD45.1^+^ and CD45.2^+^ cells in neutrophils (F), T cells (G) and B cells (H) in PB. ns, not significant; *P<0.05, **P<0.005, ***P<0.0005, ****P<0.0001 (unpaired, two-tailed Student's *t*-test). WT, *n*=3; *Zbtb33^−/y^*, *n*=3; *n* denotes the number of biological replicates.

Collectively, these results suggested that *Zbtb33*-deficient HSPCs possessed reduced self-renewal capacity (rather than an enhanced capacity) and could not drive CH.

### Loss of *Zbtb33* results in elevated neutrophils in PB in response to inflammatory stimulus

Under physiological conditions, *Zbtb33* deficiency did not cause significant hematopoietic alterations. To further investigate the hematopoietic responses to external stimuli, we examined the effects of *Zbtb33* deletion under bacterial inflammation induced by lipopolysaccharide (LPS) and non-bacterial enteritis mediated by DSS (Fig. 4A,C).

**Fig. 4.**
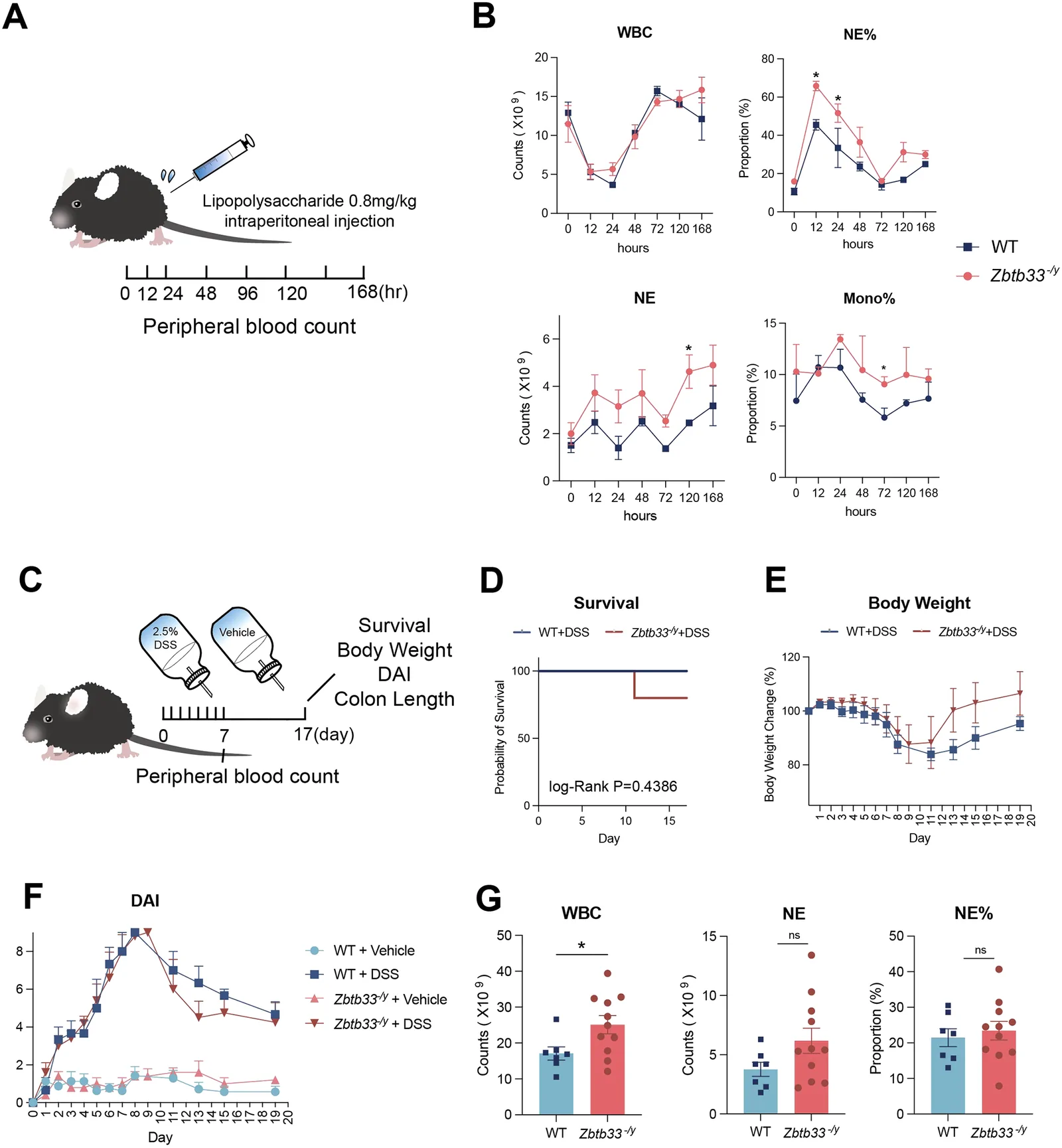
**Role of *Zbtb33* upon inflammatory challenge.** (A) Schematic diagram of an inflammatory model using lipopolysaccharide injection. (B) Changes in PB cells at the indicated time points. Mono%, monocyte cell proportion; NE, absolute neutrophil count; NE%, neutrophil proportion; WBC, white blood cells. (C) Schematic diagram of another inflammatory challenge model using dextran sulfate sodium (DSS). (D) Survival during a DSS-challenge cycle (induction phase and recovery phase). (E) Body weight changes during an entire DSS-challenge cycle. (F) Disease activity index (DAI) changes during a DSS-challenge cycle. (G) PB cell counts at 7 days post-DSS feeding. ns, not significant; *P<0.05 (unpaired, two-tailed Student's *t*-test). WT, *n*=7; *Zbtb33^−/y^*, *n*=11; *n* denotes the number of biological replicates.

Upon LPS injection, both *Zbtb33*-KO and WT mice exhibited a similar trend in WBC count changes, characterized by an initial decrease (0-24 h) followed by an increase (after 24 h). NE% peaked at 12 h post injection and declined continuously until 72 h, reaching similar minimal levels in both groups. However, during the acute inflammatory response phase (12-24 h), *Zbtb33*-deficient mice displayed a significantly higher NE% than did WT mice, indicating a more pronounced inflammatory response (Fig. 4B, *P*<0.05). Additionally, NE remained elevated in *Zbtb33*-KO mice throughout the observation period. During the recovery stage post LPS administration, NE continued to rise in *Zbtb33*-KO mice, whereas the WT group exhibited stable or fluctuating NE levels. These results suggest that *Zbtb33* deficiency enhances neutrophil differentiation or mobilization in response to inflammatory stimuli (Fig. 4B, *P*<0.05). Monocyte proportions (Mono%) also remained significantly higher in *Zbtb33*-KO mice than those in WT mice at any time point after 24 h post injection, with the largest difference observed at 72 h (Fig. 4B, *P*<0.05). No significant changes were noted in other hematological parameters (Fig. S2A).

For the DSS-induced enteritis model, four groups were studied: *Zbtb33*-deficient mice and WT mice treated with DSS or vehicle (Fig. 4C). No significant differences were observed in survival rates, body weight changes or disease activity index (DAI) scores between *Zbtb33*-KO and WT mice with DSS treatment (Fig. 4D-F). PB analysis on the final day of DSS treatment revealed significant higher WBC counts in *Zbtb33*-KO mice than those in WT mice (Fig. 4G; Fig. S2B, *P*<0.05), with elevated NE% and NE consistent with the LPS-induced findings. Moreover, colon length is an indicator of colitis severity (Lewis et al., 2023; Tian et al., 2019). After the recovery period following DSS treatment, the colons of *Zbtb33*-KO mice did not demonstrate significant shortening (Fig. S2C, *P*<0.05). Spleen weight also did not show significant differences between groups (Fig. S2D).

In summary, *Zbtb33* deficiency enhances neutrophil levels during inflammatory responses, with elevated neutrophil counts persisting even during the recovery phase. However, this effect does not significantly impact the overall progression or severity of inflammatory conditions, suggesting that *Zbtb33* deficiency modulates neutrophil dynamics but is insufficient to drive disease outcomes.

### Loss of *Zbtb33* does not enhance or promote the effects of *Tet2* LOF-mediated CH

CH is a complex consequence of interactions between multiple genetic drivers and environmental factors (Evans and Walsh, 2023). *ZBTB33*, as a candidate CH driver, encodes the transcription factor ZBTB33/KAISO, which has been reported as a methylated DNA reader (Bartels et al., 2011). *Tet2*, a commonly CH-associated gene, is closely linked to DNA methylation (Izzo et al., 2020; Belizaire et al., 2023). Previous studies, including our own (He et al., 2025), have shown that *Tet2* LOF mutations promote HSPC self-renewal and mediate myeloid-skewing differentiation (Nakauchi et al., 2022). Using the BloodSpot database (Gíslason et al., 2023), we found that *ZBTB33* expression is elevated in early and intermediate stages of myeloid differentiation, such as CMPs, early and late promyelocytes, while *TET2* expression increases during late myeloid differentiation, especially in polymorphonuclear neutrophils (Fig. S3A).

To investigate potential genetic interactions between *Zbtb33* and *Tet2*, we crossed *Zbtb33*-deficient mice with mice harboring the *Tet2* LOF mutations (Fig. 5). Four groups of mice were studied: *Zbtb33* mutant mice (*Zbtb33*^−*/y*^), *Tet2* LOF heterozygous mutant mice (*Tet2*^+/−^), *Zbtb33* and *Tet2* double-mutant mice (*Zbtb33*^−*/y*^*; Tet2*^+/−^), and WT littermate controls. *Tet2*-LOF mice exhibited significantly elevated neutrophils (NE and NE%) and decreased lymphocytes compared to WT mice, consistent with the myeloid expansion mediated by *Tet2* mutations. In *Zbtb33^−/y^; Tet2^+/−^* mice, we observed elevated WBCs, platelets, NE and NE%, alongside reduced LY%, compared to that in *Zbtb33^−/y^* mice (Fig. 5A). However, no significant differences were noted between *Tet2^+/−^* and *Zbtb33^−/y^; Tet2^+/−^* mice, indicating that *Zbtb33* mutation had minimal effects on PB parameters under *Tet2* LOF conditions. Spleen weights in *Zbtb33^−/y^; Tet2*^+/−^ mice showed significant increases compared to those in *Zbtb33^−/y^* littermates, but no differences when compared to those in the *Tet2^+/−^* mice (Fig. 5B).

**Fig. 5.**
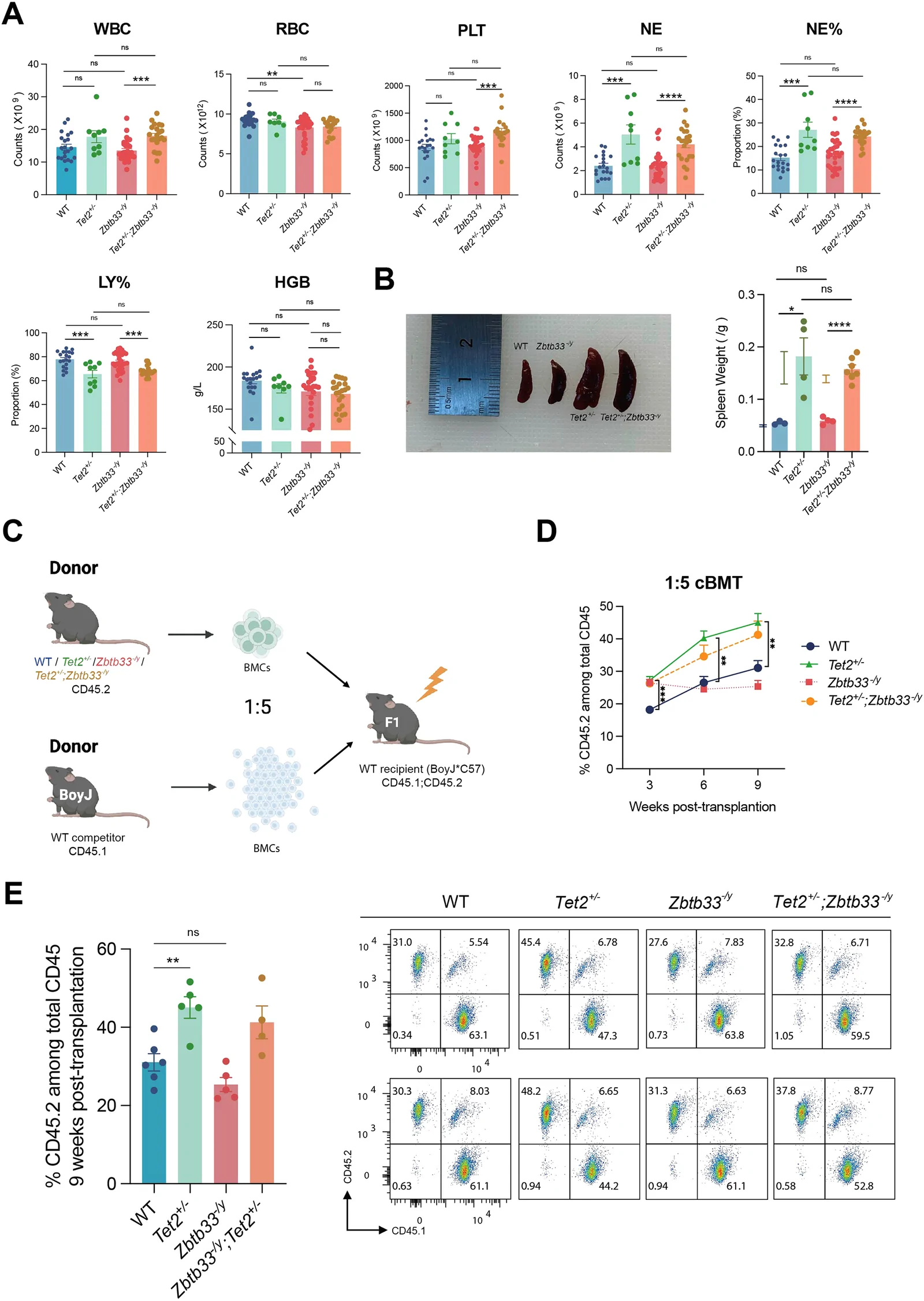
**Absence of *Zbtb33* does not enhance or diminish the effects of *Tet2* loss of function.** (A) Blood cell counts in WT, *Tet2^+/−^*, *Zbtb33^−/y^* and compound mutants *Tet2^+/−^; Zbtb33^−/y^*. HGB, hemoglobin; LY%, lymphocyte proportion; NE, absolute neutrophil count; NE%, neutrophil proportion; PLT, platelets; RBC, red blood cells; WBC, white blood cells. (B) Representative images and weight measurement of spleens in the indicated genotypes. (C) Schematic diagram of cBMT assay in mice of the indicated genotypes. Mixing ratio: 1:5. (D) PB chimerism analysis at the indicated time points with four different genetic background donors. (E) Representative flow cytometry profiles and quantification results of PB chimerism at monitoring endpoints (9 weeks post cBMT). ns, not significant; *P<0.05, **P<0.005, ***P<0.0005, ****P<0.0001 (unpaired, two-tailed Student's *t*-test). WT, *n*=20; *Tet2^+/−^*, *n*=9; *Zbtb33^−/y^*, *n*=28; *Tet2^+/−^*; *Zbtb33^−/y^*, *n*=22; *n* denotes the number of biological replicates.

We performed cBMT assays at a 1:5 ratio to assess the clonal expansion of HSPCs with *Zbtb33* and *Tet2* mutations (Fig. 5C)*.* Recipients of *Tet2*^+/−^ BMCs showed significantly higher PB chimerism than did WT donors, confirming *Tet2*-LOF mutation-mediated clonal expansion. In contrast, *Zbtb33^−/y^* BMCs exhibited the lowest chimerism. Interestingly, although double-mutant BMCs displayed greater clonal expansion than did WT or *Zbtb33*^−*/y*^ BMCs, their expansion was moderately reduced compared to that in *Tet2*^+/−^ BM donors (Fig. 5D,E). These findings suggested that loss of *Zbtb33* did not enhance or promote *Tet2*-driven CH.

Considering the established role of ZBTB33/KAISO in DNA damage responses in a *P53*-dependent manner (Di et al., 2014), we also examined the interplay between *Zbtb33* and *P53*, another key driver of CH. Analysis of the BloodSpot database revealed that *P53*, similar to *Zbtb33*, is highly expressed in myeloid lineages of HSPCs, including CMPs, granulocyte-macrophage progenitors, and early and late promyelocytes (Fig. S3A). We generated *Zbtb33*^−/*y*^*; P53^+/−^* mice to assess whether *Zbtb33* deficiency influenced *P53* mutation-driven CH. As expected, *P53* mutations caused myeloid expansion, with significant increases in WBCs, NE% and NE. However, no discernible differences in PB indices were observed between *Zbtb33*^−/*y*^*; P53^+/−^* mice and those with a single *P53* mutation, indicating that *Zbtb33* deficiency does not significantly alter the hematological changes induced by *P53* mutation (Fig. S3B).

To investigate the impact of *Zbtb33* deficiency on hematopoiesis in an inflammatory context caused by genetic mutations, we crossed *Zbtb33*-KO mice with *Pstpip2*-KO mice, a model that exhibits sterile inflammation (Geng et al., 2024). This model mimics the clinical features of chronic multifocal osteomyelitis and shows hallmarks of auto-inflammatory disease, including extramedullary hematopoiesis and joint swelling. We conducted a 3-month observational study on the four groups of mice: WT mice, *Zbtb33*-deficient mice (*Zbtb33*^−/*y*^), *Pstpip2* mutant mice (*Pstpip2*^−/*−*^), and *Zbtb33* and *Pstpip2* double-mutant mice (*Pstpip2*^−/*−*^; *Zbtb33*^−/*y*^). Through tracking and scoring the number of swollen hind paws over time, we observed a progressive escalation of osteomyelitis symptoms, as indicated by increasing count of swollen paws with age in *Pstpip2* mutant background. Notably, no significant differences in paw swelling were observed between the *Pstpip2*^−/*−*^*; Zbtb33*^−/*y*^ double-mutant and *Pstpip2^−/−^* single-mutant mice (Fig. S3C). PB analysis of 3-month-old mutant mice showed a significant increase in neutrophil count and proportion in *Pstpip2*^−/*−*^ mice compared to that in WT mice. However, no notable differences were observed between double-mutant mice and *Pstpip2* single-mutant mice. These results indicated that *Zbtb33* deficiency did not exert a substantial influence on the myeloid expansion driven by the *Pstpip2* mutation (Fig. S3D).

### Loss of *Zbtb33* in the tumor microenvironment promotes tumor growth

To investigate the potential molecular alterations in the hematopoietic system caused by *Zbtb33* deficiency, we performed bulk RNA-seq on BMCs from WT mice and *Zbtb33*-deficient mice (*n*=4 per group). Differentially expressed gene (DEG) analysis identified the top ten genes in *Zbtb33*-KO mice (Fig. 6A). Among the upregulated genes, *Hspb8*, which encodes small heat shock protein B8, stood out. *Hspb8* exhibits dual roles in tumorigenesis, either promoting or suppressing tumor growth depending on the tumor type (Cristofani et al., 2022). Previous studies have shown that *Hspb8* mediates autophagy in melanoma, thereby inhibiting tumor cell growth and, thus, exerting anti-tumor effects. The heatmap also revealed a significant increase in the expression of *Igkv4* (an immunoglobulin kappa variable gene) in *Zbtb33*-deficient BMCs, suggesting immune dysregulation in the bone marrow. Additionally, *Platr22*, a long non-coding RNA known to influence cell fate and stem cell pluripotency through epigenetic regulation (Yan et al., 2021), was markedly upregulated in *Zbtb33*-deficient mice. Another upregulated gene, *Apob*, which encodes apolipoprotein B protein, is involved in the transport of low-density lipoprotein and very-low-density lipoprotein, and is closely associated with cardiovascular disease (Bilgic et al., 2024). In contrast, significantly downregulated genes included *Nign1*, *Ren1* and *Ren2*, which are linked to the angiotensin-converting enzyme inhibitor pathway. These findings suggest that *Zbtb33* deficiency regulates tumorigenesis, immune responses, cell fate, stem cell pluripotency and cardiovascular diseases (Fig. 6A).

**Fig. 6.**
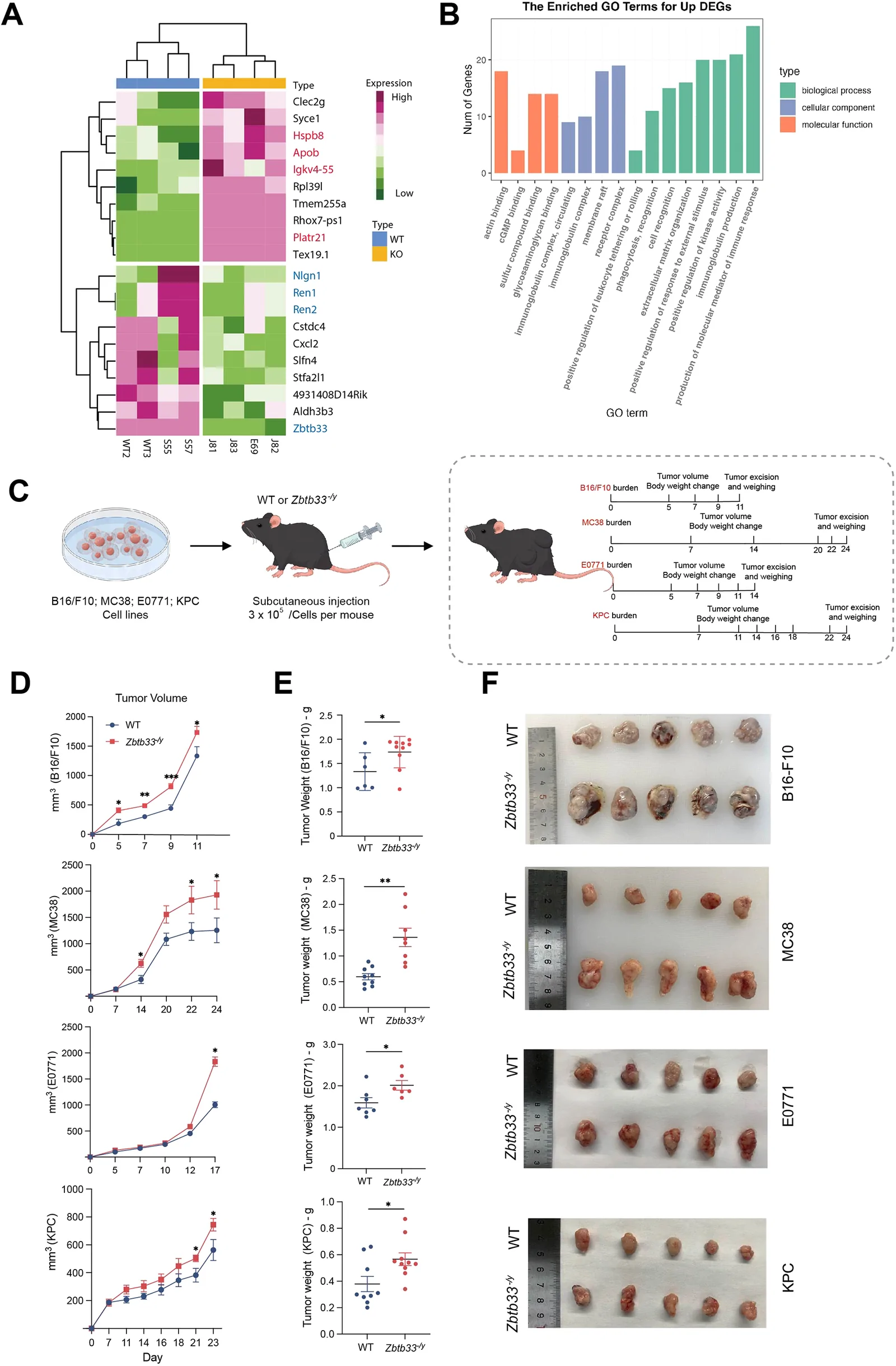
**Loss of *Zbtb33* in the tumor microenvironment exacerbates tumor growth.** (A) RNA-sequencing analysis of *Zbtb33*-KO BMCs. Heatmap highlights differentially expressed genes (DEGs). Pink indicates upregulated genes; green indicates downregulated genes. (B) The top upregulated Gene Ontology (GO) pathways in *Zbtb33*-KO BMCs primarily included immune-related processes. (C) Schematic diagram of subcutaneous tumor model using B16/F10, MC38, E0771 and KPC cell lines. (D) Tumor volume was measured at the indicated time points in *Zbtb33^−/y^* and WT mice subcutaneously inoculated with B16/F10, MC38, E0771 or KPC cells. B16/F10: WT, *n*=6; *Zbtb33^−/y^*, *n*=10. MC38, WT, *n*=9; *Zbtb33^−/y^*, *n*=8. E0771: WT, *n*=7; *Zbtb33^−/y^*, *n*=6. KPC, WT, *n*=9; *Zbtb33^−/y^*, *n*=10. *n* denotes the number of biological replicates. (E) Tumor weight of excised tumors at the experimental endpoint. (F) Representative images of excised tumors at the experimental endpoint. *P<0.05, **P<0.005, ***P<0.0005 (unpaired, two-tailed Student's *t*-test).

Gene Ontology (GO) enrichment analysis highlighted upregulated immune response-related pathways in *Zbtb33*-deficient BMCs, including the production of molecular mediators of immune response, immunoglobulin production and circulating, and receptor complex formation (Fig. 6B). Accordingly, we evaluated any positive roles of *Zbtb33* in cancer immunity using four murine tumor-bearing models: B16/F10 melanoma, MC38 colorectal carcinoma, E0771 mammary carcinoma and KPC pancreatic ductal adenocarcinoma (Fig. 6C). Strikingly, across all four tumor-bearing models, *Zbtb33*-KO mice exhibited greater tumor burden at the experimental endpoint than did WT controls (Fig. 6D-F, *P*<0.05). The consistency of this phenotype across distinct tumor types indicates that *Zbtb33* functions as an anti-tumor factor within the tumor immune microenvironment.

To further delineate whether the anti-tumor function of *Zbtb33* originates from hematopoietic or non-hematopoietic cell lineages, we generated additional four groups of bone marrow chimeric mice – WT→WT, *Zbtb33*-KO→WT, WT→*Zbtb33*-KO and *Zbtb33*-KO→*Zbtb33*-KO (donor→recipient) – and subjected them to subcutaneous MC38 tumor inoculation with serial tumor volume measurements (Fig. 7A). One-way ANOVA across all four groups revealed a significant difference beginning at day 26 post tumor inoculation (*P*=0.024), with significance increasing at days 30 (*P*=0.0008), 35 (*P*=0.003) and 38 (*P*=0.002). Post-hoc Tukey honest significant difference (HSD) analysis demonstrated that *Zbtb33*-KO→WT exhibited significantly greater tumor burden than did WT→WT controls at days 30, 35 and 38 (*P*=0.025, *P*=0.026 and *P*=0.036, respectively). In contrast, WT→*Zbtb33*-KO showed no significant difference in tumor growth compared to that in WT→WT controls at any time point examined (*P*>0.50 at all post-day 26 assessments), suggesting that *Zbtb33* deficiency in the stromal compartment does not substantially alter the tumor microenvironment's permissiveness to tumor growth. Meanwhile, *Zbtb33*-KO→*Zbtb33*-KO chimeras displayed greater tumor burden than did WT→*Zbtb33*-KO chimeras at days 30 and 38 (*P*=0.044 and *P*=0.016, respectively), further indicating that additional *Zbtb33* deficiency in the hematopoietic compartment further exacerbates tumor growth even when the stromal compartment also lacks *Zbtb33*. Analysis of the area under the tumor growth curve (AUC), integrating tumor burden across the full experimental time course, confirmed that the *Zbtb33*-KO→*Zbtb33*-KO group exhibited a significantly greater cumulative tumor burden than did WT→WT controls (one-way ANOVA, *P*=0.028; Tukey HSD, *P*=0.015), whereas no significant difference in AUC was observed among *Zbtb33*-KO→WT, WT→*Zbtb33*-KO and WT→WT groups (all *P*>0.22) (Fig. 7B). At the experimental endpoint, tumor weight differed significantly across the four chimeric groups (one-way ANOVA, *P*=0.021). Specifically, *Zbtb33*-KO→WT chimeras (1.08±0.50 g) showed a numerically higher tumor weight than WT→WT controls (0.64±0.17 g). Notably, *Zbtb33*-KO→*Zbtb33*-KO chimeras (1.33±0.43 g) also presented a higher tumor weight than did WT→*Zbtb33*-KO chimeras (0.86±0.32 g, *P*=0.030) whereas WT→*Zbtb33*-KO chimeras showed no significant difference from WT→WT controls (*P*=0.782), consistent with the tumor growth kinetics data (Fig. 7C).

**Fig. 7.**
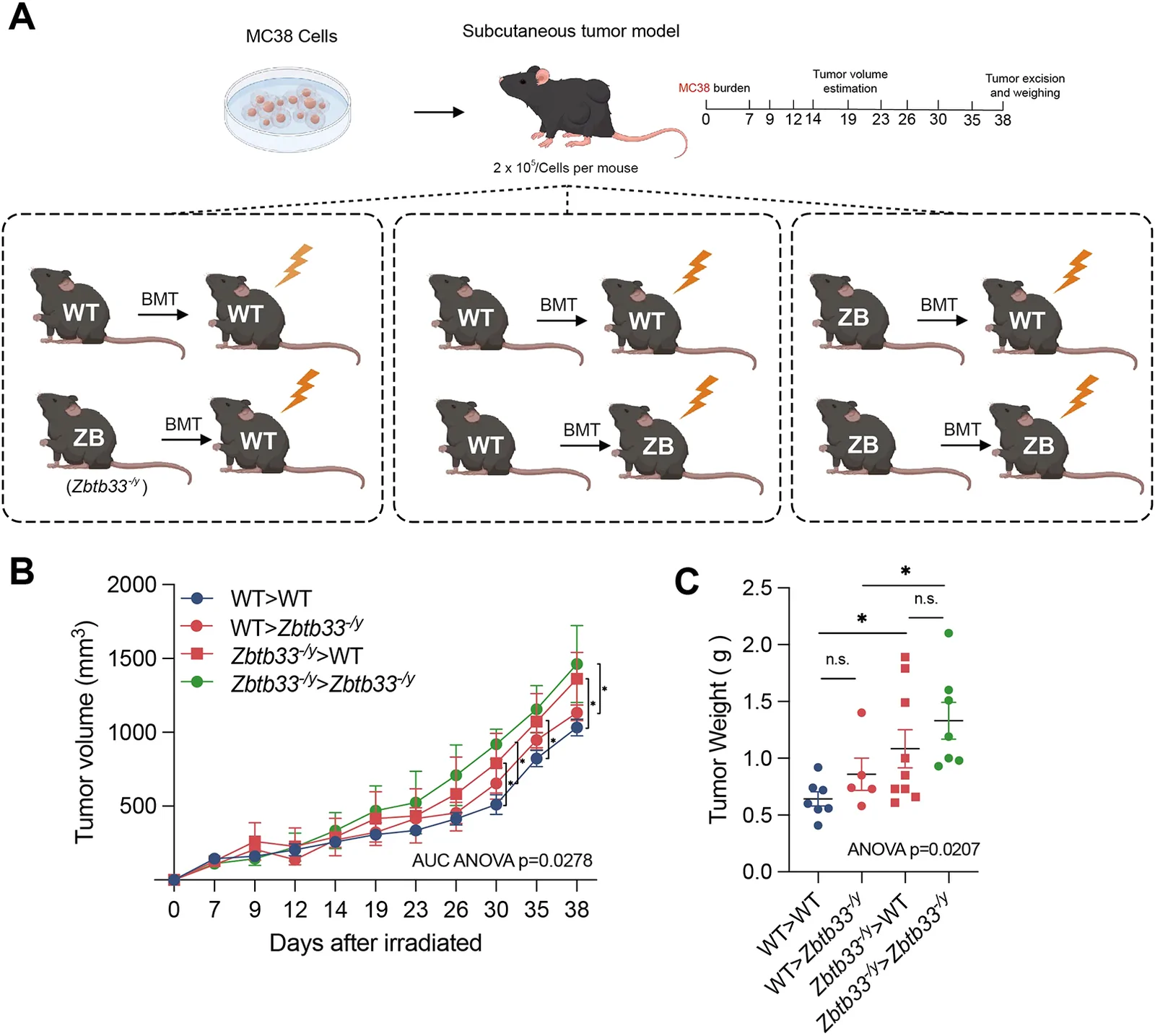
**Hematopoietic *Zbtb33* deficiency accelerates tumor growth in bone marrow chimeric mice.** (A) Schematic diagram illustrating the experimental design for bone marrow chimera generation and subcutaneous tumor inoculation. Donor BMCs from WT or *Zbtb33*-KO mice were transplanted into lethally irradiated recipient mice, followed by MC38 subcutaneous tumor challenge after hematopoietic reconstitution. BMT, bone marrow transplantation. (B) Tumor growth curves of WT→WT, WT→*Zbtb33*-KO, *Zbtb33*-KO→WT and *Zbtb33*-KO→*Zbtb33*-KO chimeric mice following MC38 subcutaneous inoculation (donor→recipient). Tumor volume was measured at the indicated time points. (C) Tumor weight of excised tumors from *Zbtb33*-KO→WT and *Zbtb33*-KO→*Zbtb33*-KO chimeric mice at the experimental endpoint. AUC, area under the tumor growth curve. Data are presented as mean±s.e.m. n.s., not significant; *P<0.05 (one-way ANOVA with Tukey's HSD post-hoc test).

Taken together, these results indicate that the impaired anti-tumor response associated with *Zbtb33* deficiency is primarily attributable to the hematopoietic compartment. However, given that the present study did not include formal immune cell depletion experiments, the specific effector cell populations responsible for this phenotype cannot be conclusively determined from these data alone. Further studies are required to delineate the details of *Zbtb33*-related immune regulation.

## DISCUSSION

CH is defined by age-related expansion of premalignant HSCs with somatic mutations in certain CH-driver genes (*TET2* and *P53*). Intriguingly and importantly, this phenomenon is associated with the development and progression of various diseases, encompassing both hematological and non-hematological conditions such as Alzheimer's disease, cardiovascular diseases, chronic inflammation (Bouzid et al., 2023) and hematopoietic malignancies, particularly myeloid neoplasms (Gillis et al., 2017). Mutations in *DNMT3A*, *TET2* and *P53* (characterized by LOF) and in *JAK2* (characterized by gain of function), are well-established driver mutations of CH (Steensma, 2018), supported by clinical evidence and experimental validation. Those CH-associated mutations increase the risk of hematological malignancies and various chronic diseases associated with CH. However, the full spectrum of mutations that drive CH have not yet been fully elucidated, and there is an urgent need for experimental validation of potential candidate driver mutations. Studies from clinical hematology and mouse genetic models are important for validating CH drivers, providing a comprehensive understanding of the genetic underpinnings of this condition and its associated diseases.

In 2021, mutations in *ZBTB33* were first reported as a novel candidate driver for CH through the integration of DNA-sequencing datasets from TOPMed and ExAC (Beauchamp et al., 2021). By 2024, *ZBTB33* had been reaffirmed as a ‘new fitness-inferred driver’ of CH, based on analyses of whole-blood somatic mutations and selection from single-cell-derived hematopoietic colonies (Bernstein et al., 2024). Here, we conducted an in-depth investigation of the mouse homolog of *ZBTB33*, *Zbtb33*, using mouse models. However, our experimental analyses did not substantiate the notion that the loss of *Zbtb33* drives CH in the mouse models. Functional analyses, including cBMT experiments, indicated that *Zbtb33* deletion did not promote the renewal and expansion associated with inducing CH, as tested at mixing ratios of 1:1 or 1:5. These findings are consistent with a recent study identifying *DNMT3A*, *TET2*, *ASXL1* and *KMT2D* as the predominant driver genes of CH, in which *ZBTB33* was not reported as a recurrent driver (Xu et al., 2026 preprint). In an analysis of cancer risk-related AML mutations, *ZBTB33* was not screened (Cheek et al., 2026). Likewise, *ZBTB33* was not included in the panel of CHIP driver genes analyzed by Yoshida et al. (2026). *ZBTB33* was also not included in the panel of screened genes in a lineage-tracing assay that profiled hematopoietic clones based on well-established CH driver mutations (Yoshizato et al., 2025). Taken together, our results suggest that *Zbtb33* LOF mutations are insufficient to confer a clonal advantage to HSCs. It is worth noting, however, that our findings appear to contrast with those reported by Beauchamp et al. (2021), who conducted minimal experimental work utilizing CRISPR/Cas9-based gene KO of *Zbtb33* in mouse primary HSCs followed by transplantation. The discrepancy between studies may be attributable to methodological differences, including variations in experimental systems, KO efficiency, transplantation conditions or the genetic background of the mouse models employed. Future studies with standardized experimental approaches will be necessary to reconcile these conflicting observations.

Our previous studies, supported by corroborating evidence from other research, indicated that the LOF of the DNA demethylase *Tet2*, a pivotal driver in CH, enhances the self-renewal ability of HSCs and predominantly steers their differentiation towards myeloid lineages (Agrawal et al., 2022; Evans et al., 2020). Mutations in the DNA damage repair gene *P53* represent another prevalent driver category in CH. *P53* has been implicated in regulating ZBTB33/KAISO expression (Di et al., 2014; Choi et al., 2019; Koh et al., 2015). We conducted a series of experiments to investigate the potential synergistic effects of *Zbtb33* mutations and *Tet2* or *P53* mutations in CH. However, neither the *Zbtb33^−/y^; Tet2^+/−^* nor *Zbtb33^−/y^; P53^+/−^* mouse models exhibited accelerated progression of CH compared to that of their respective *Tet2^+/−^* or *P53^+/−^* single-mutant controls. Similarly, *Zbtb33* deficiency was not sufficient to enhance or alter the phenotypes in well-established chronic osteomyelitis models mediated by *Pstpip2* mutations. This could indicate that the synergistic effects of *ZBTB33* with *TET2*, *P53* and *PSTPIP2* in hematopoiesis are limited, and that the impact of *ZBTB33* in CH is comparatively marginal when compared with that of other well-characterized driver genes.

Under normal physiological conditions, *Zbtb33^−/y^* mice did not exhibit abnormalities. However, we were intrigued by the possibility that these mutant mice might exhibit aberrant phenotypes when confronted with pathogens or inflammation. Previous studies have indicated that overexpression of ZBTB33/KAISO can promote intestinal inflammation, which is associated with altered p120ctn function and potentially linked to the disruption of the intestinal immune barrier and increased intestinal permeability (Robinson et al., 2019). In our study, we assessed the responses of *Zbtb33*-null mice to LPS and DSS challenges. In our experimental setup, these two different external inflammation stimuli did not affect mouse survival. Moreover, upon LPS challenge or DSS treatment, the phenotypic indicators suggested that the phenotypes of *Zbtb33* mutants were largely indistinguishable from those of WT controls, with the exception of a slight increase in NE% (Fig. 4) in PB of *Zbtb33^−/y^* mice compared to that in PB of WT mice. These findings suggested a minimal or dispensable role of *Zbtb33* in inflammation resolution.

In addition, the role of *Zbtb33* in oncogenesis has been a subject of controversy (Pierre et al., 2019), with evidence suggesting its capacity to act as both transcription activator and repressor (Wiese et al., 2013). A previous study identified *Zbtb33* as a tumor suppressor (Buck-Koehntop et al., 2012). Its repression of key target genes such as *Matrilysin* (also known as *Mmp7*), *Siamois* and *Wnt11* has been implicated as a crucial mechanism in its antitumorigenic activity (Dai et al., 2009; Feng, 2018; Van Roy and McCrea, 2005). Our findings revealed that hematopoietic deletion of *Zbtb33* accelerated tumor growth across four syngeneic murine tumor models: B16/F10 melanoma, MC38 colorectal carcinoma, E0771 mammary carcinoma and KPC pancreatic ductal adenocarcinoma. This consistent phenotype across distinct tumor types indicates that the absence of *Zbtb33* broadly reshapes anti-tumor response in a tumor-type-independent manner, supporting its function as a tumor suppressor gene. Bone marrow chimera experiments further established that this effect is predominantly mediated by hematopoietic-derived cells rather than non-hematopoietic stromal components, pointing to a hematopoietic cell-intrinsic role of ZBTB33 in limiting tumor growth. Nevertheless, the precise downstream effector pathways and the specific immune cell subsets – including T cells, B cells and innate immune populations – responsible for this phenotype remain to be determined, and warrant further in-depth mechanistic investigation using single-cell transcriptomic profiling and lineage-specific conditional KO approaches (Pan et al., 2019).

In summary, this study provides experimental results to suggest the following: (1) similarly to *Tet2* and *P53*, *Zbtb33* is broadly expressed across various hematopoietic cell types; (2) functionally, *Zbtb33^−/y^* mutant mice (male) appear physiologically normal and do not exhibit any overt abnormalities under normal conditions; (3) the loss of *Zbtb33* in HSCs does not promote CH; (4) when challenged with LPS or DSS, or when bred with *Pstpip2^−/−^* mice, *Zbtb33^−/y^* mice do not manifest abnormalities in inflammation resolution; and (5) *Zbtb33^−/y^* mice exhibit consistently greater tumor burden across multiple tumor models, suggesting that ZBTB33 contributes to tumor growth control through hematopoietic rather than non-hematopoietic lineages. Given that our findings are based solely on the *Zbtb33-*KO mouse model, further experimental analysis using human BMCs is necessary to determine the inclusion or exclusion of *ZBTB33* in the gene-mutation panel tests for CH and MDS. This additional research will help to elucidate the role of ZBTB33 in human disease contexts and guide clinical decision making.

### Conclusion

This study shows that *Zbtb33* deficiency does not drive CH or enhance the expansion of HSCs, even in combination with *Tet2* or *P53* mutations. Although ZBTB33 does not play a direct role in CH, hematopoietic loss of *Zbtb33* accelerates tumor growth across multiple tumor models. These findings suggest that ZBTB33 plays a limited role in hematopoiesis but contributes to tumor growth control through hematopoietic lineages, warranting further investigation into the specific cell types and mechanisms involved.

## MATERIALS AND METHODS

### Generation of *Zbtb33*-deficient transgenic mice

CRISPR/Cas9 technology was employed to target the *Zbtb33* gene, located on the X chromosome of C57BL/6 mice, for KO. A specific sgRNA was designed, and high-throughput electroporation of fertilized eggs was conducted to generate *Zbtb33*-KO mice, with assistance from Cyagen US, Inc. C57BL/6 (CD45.2^+^) and BoyJ (CD45.1^+^) mice were purchased from Cyagen US, Inc. and used in the experiments. All animals were housed under specific pathogen-free conditions with a 12-h light/dark cycle at Tianjin Medical University. The study included younger adult mice aged 8 weeks and older mice aged 6-12 months. The animal protocols were provided by Cyagen US, Inc., and the experiment was conducted in accordance with the regulations of the Animal Ethics Committee of Tianjin Medical University, with their approval.

### Genotyping and PCR

Transgenic pups were identified through PCR analysis of DNA extracted from tail biopsies. The primer pair used specific to sequences within the murine *Zbtb33* gene was as follows: forward primer (F1), 5′-TATGGGTCCTTTGCACTTCAGAT-3′; reverse primer (R1), 5′-CACTGAAAACTTCATCTTCACACCT-3′. PCR was conducted following standard protocols.

### Complete blood count procedure

Blood was collected from the outer canthus of mice using a capillary glass tube (Kimble) and transferred into an EDTA-containing Eppendorf tube. The EDTA and blood were thoroughly mixed for 30 s using a mixing device (Kylin-Bell). Subsequently, the proportions of blood cells were determined using an automated complete blood count machine (SHENGCHENYILIAO).

### Mouse tissue collection

Mice were euthanized by CO_2_ asphyxiation, and the abdominal cavity was exposed. Spleens, colons and tumors were removed and immediately placed in pre-cooled PBS (Solarbio) on ice for subsequent investigation. BMCs were harvested from the femurs and tibias of mice by flushing the bone marrow cavity with sterile PBS. The cell suspension was filtered through a 70 μm cell strainer (BD Biosciences) and washed twice with PBS.

### Flow cytometry analysis of PB cells and BMCs

Purified murine PB mononuclear cells were stained for the following surface markers: CD3-PE (100206, BioLegend; 1:100), CD19-APC (152410, BioLegend; 1:100), GR1-PE (108408, BioLegend; 1:100), Mac-1/CD11b-FITC (101206, BioLegend; 1:100). The hematopoiesis stem progenitor cell subpopulations were characterized with the following staining strategies: cKit-APC (105812, BioLegend; 1:100), Sca1-APC-Cy7 (108126, BioLegend; 1:100), CD150-PE-Cy5.5 (115912, BioLegend; 1:100), CD48-PE-Cy7 (103424, BioLegend; 1:100). For myeloid progenitor cell subsets, the following antibodies were used: anti-cKit-APC (105812, BioLegend; 1:100), anti-Sca1-APC-Cy7 (108126, BioLegend; 1:100), anti-CD34-FITC (11-0341-85, eBioscience; 1:100), anti-CD16-PE-Cy7 (101318, BioLegend; 1:100). Flow cytometry was performed utilizing a BD LSRFortessa analyzer, and data were analyzed using FlowJo software (version 10.8.1, FlowJo LLC). Gating strategies were established based on forward and side scatter to remove debris and doublets, and specific cell populations were identified based on their surface marker.

### Competitive bone marrow transplantation

BMCs were harvested from either WT or *Zbtb33*-KO mice with a C57BL/6 background (CD45.2^+^). First-generation (F1) mice (CD45.1^+^; CD45.2^+^) were generated by crossing C57BL/6 WT mice (CD45.2^+^) with BoyJ mice (CD45.1^+^). F1 recipient mice underwent lethal irradiation with doses of 10 Gy and 7 Gy, separated by a 4-h interval. BMCs from WT or *Zbtb33*-KO mice (CD45.2^+^) were mixed with BoyJ competitor cells (CD45.1^+^) at ratios of 1:1 and 1:5 and were injected into the tail vein of F1 (CD45.1^+^; CD45.2^+^) mice 24 h post irradiation. The chimerism of CD45.2^+^ cells in the PB of recipient mice was evaluated every 3 or 4 weeks during the transplantation period. For secondary transplantations, BMCs derived from the first recipients were transplanted into each lethally irradiated recipient. PB chimerism was measured every 4 weeks, and bone marrow chimerism was observed at the endpoint of the observation period.

In additional cBMT experiments to assess the effects of *Zbtb33* on hematopoiesis in *Tet2*-mutant mice, donors included *Zbtb33^−/y^/Tet2^+/−^* single- and double-mutant mice and WT mice, with F1 mice serving as recipients.

### Mouse inflammatory stimulus experiment

LPS, derived from *Escherichia coli* (L2880, Sigma-Aldrich) was administered intraperitoneally to mice at a dose of 0.8 mg/kg. The control group received an equal volume of PBS as the vehicle. PB samples from these mice were collected and sequentially monitored at 12, 24, 48, 96, 120 and 168 h post administration.

DSS was obtained from MP Biomedicals and dissolved in sterile water to prepare a 2.5% solution. Mice were randomly assigned to four groups: *Zbtb33* mutant mice fed with DSS (*Zbtb33^−/y^*+DSS), WT mice fed with DSS (WT+DSS), *Zbtb33* mutant mice fed with vehicle (*Zbtb33^−/y^*+vehicle) and WT mice fed with vehicle (WT+vehicle). For the initial 7 days, the experimental groups (WT+DSS; *Zbtb33^−/y^*+DSS) were fed 2.5% DSS to induce non-bacterial colitis, followed by a 10-day recovery phase during which they were fed the vehicle solution. Meanwhile, the control groups (WT+vehicle; *Zbtb33^−/y^*+vehicle) were fed the vehicle solution throughout the entire period. After completing the entire cycle, the survival rate, body weight changes, DAI and colon length were measured and recorded for each group.

### DAI for enteritis model

The DAI is an important indicator utilized for evaluating the severity of colitis. DAI scores include factors such as changes in body weight, stool consistency and the presence of occult blood in stools (Liu et al., 2020; Yan et al., 2018). The fecal occult blood test was performed using an o-toluidine-based test kit, along with its instruction manual, provided by Beijing Leagene Biotechnology Co., Ltd. In the o-toluidine-based fecal occult blood test, the reagent reacts chemically with blood in the stool, causing a color change, typically to blue or blue-green, when blood is present. The speed and intensity of the color change indicate the severity of occult bleeding (Table 1).

**Table 1.** Evaluation indicators of the disease activity index

Scores	0	1	2	3	4
Weight change (%)	No change	1-5%	5-10%	10-20%	>20%
Stool consistency	Normal	Loose stool	Watery diarrhea	Slimy diarrhea	Diarrhea
Fecal occult blood	No color change within 2 min	Gradually changes from light green to green after 10 s	Initially shows as light green, gradually turning to a distinct blue-brown color	Immediately shows as blue-brown color, gradually turning to black-brown	Immediately shows blue-black-brown color

### Subcutaneous tumor formation model

B16F10 melanoma cells and MC38 colon cancer cells were kindly provided by Dr Deqin Hu (Tianjin Medical University School of Basic Medicine), E0771 breast tumor cells and KPC pancreatic ductal adenocarcinoma cells were kindly provided by Dr Dan Ye (Shanghai Medical College of Fudan University). All cell lines were authenticated by short tandem repeat (STR) profiling and tested negative for mycoplasma contamination prior to use. B16F10 melanoma cells, MC38 colon cancer cells, E0771 breast tumor cells and KPC pancreatic ductal adenocarcinoma cells were suspended in 100 μl PBS containing 50 μl Matrigel (356234, BD Biosciences). The cell suspension was mixed with Matrigel at a 1:1 ratio in a 4°C environment to a final concentration of 3×10^5^ cells/ml. Prior to inoculation, mice were weighed to ensure that their weight was between 18 and 22 g. The hair on the lower-right side of their back was shaved using a mouse hair trimmer to expose the skin, which was then disinfected with 75% ethanol. The cell suspension was gently resuspended, and 100 μl was slowly injected subcutaneously into the mouse.

Tumor growth was monitored by measuring the maximum diameter (*L*) and minimum diameter (*W*) of the tumors every 2 days with a vernier caliper. Tumor volume (*V*) was calculated using the formula (*V*=*L*×*W*^2^/2).

When the tumor volume reached ∼2000 mm^3^, all mice were humanely euthanized by exposure to CO_2_.

### Bulk RNA-seq of BMCs

BMCs were obtained from *Zbtb33^−/y^* mice and their WT littermates. RNA was isolated and extracted using an RNeasy mini kit (Qiagen) following the manufacturer's instructions. The quality and quantity of the RNA were assessed using a NanoDrop spectrophotometer (Thermo Fisher Scientific) and an Agilent 2100 Bioanalyzer (Agilent Technologies). Bulk RNA-seq was performed at Majorbio using a NovaSeq 6000 platform (Illumina). Libraries were prepared using a TruSeq RNA Sample Preparation Kit (Illumina) according to the standard protocol. The resulting libraries were sequenced on a NovaSeq 6000 platform to generate 150 bp paired-end reads.

Raw sequencing reads were processed using the fastp tool (version 0.20.0) to perform quality control and trimming. The cleaned reads were aligned to the reference genome (*Mus musculus*, GRCm38) using the STAR aligner (version 2.7.3a). Aligned reads were quantified using featureCounts (version 1.6.4) from the Subread package. The output from featureCounts was used for downstream differential expression analysis.

### DEG analysis and enrichment analysis

DEG analyses between *Zbtb33*-KO and WT mice (four mice per group) were performed using R software (version 4.2.1), R Studio and DESeq2 (version 1.36.0). Genes with *P*-values below 0.05 were considered significantly differentially expressed. GO enrichment analysis was conducted using clusterProfiler based on log2 fold change results obtained from the DEG analysis and visualized by ggplot2 R package (Yu et al., 2012).

### Statistical analysis

Statistical comparisons across multiple groups were performed using one-way ANOVA, followed by Tukey HSD post-hoc test for pairwise comparisons. For non-normally distributed data, Kruskal–Wallis test with Mann–Whitney *U* pairwise comparisons was applied. Single comparisons relied on unpaired, two-tailed Student's *t*-tests. Log-rank tests were applied for survival analyses. In general, *P*-values <0.05 were considered significant. *P*-values were calculated using GraphPad Prism 9.0 software.

## Supplementary Material

10.1242/dmm.052484_sup1 Supplementary information
