# Supplementary material for: Loss of transcription factor Zbtb33 fails to induce clonal hematopoiesis but plays a role in tumor growth
Source: Dis Model Mech. 2026 Oct 26;19(10):dmm052484. doi: 10.1242/dmm.052484 (PMC13750393; doi:10.1242/dmm.052484)
Supplement: Supplementary file 1 [file dmm-19-052484-s1.pdf]

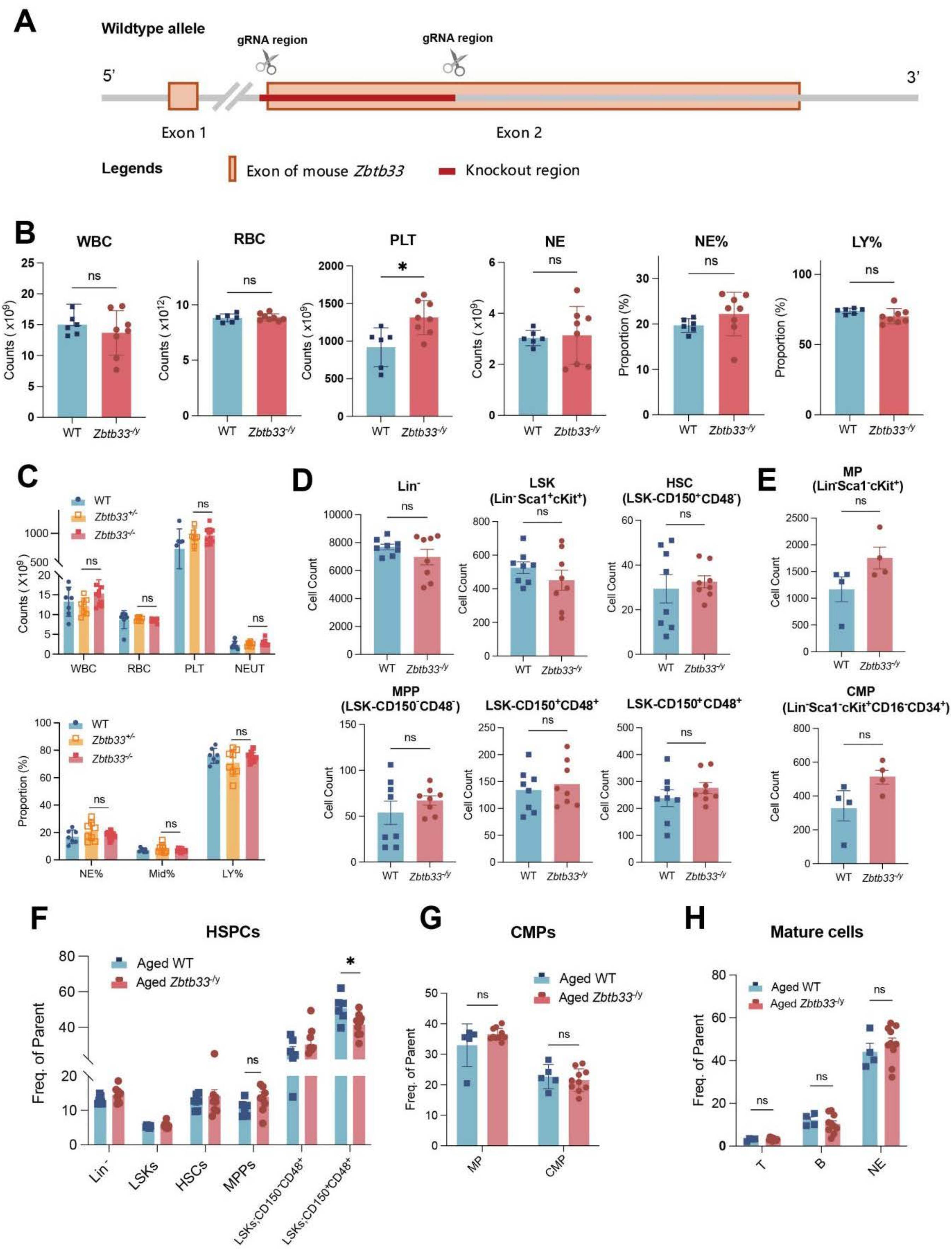

Disease Models & Mechanisms • Supplementary information

Fig. S1. related to Figure 1:

(A) Overview of the targeting strategy for generating *Zbtb33*-knockout mice. Exon2 is removed in the *Zbtb33*$^{-/y}$ mice.

(B) Bar chart showing various parameters in whole blood counts in aged *Zbtb33*-knockout mice and matched wildtype littermates. WT (n=6), *Zbtb33*$^{-/y}$ (n=8) biological repeats.

(C) Peripheral blood cell counts for *Zbtb33*$^{+/-}$, *Zbtb33*$^{-/-}$ mice and wildtype female mice. WT (n=7), *Zbtb3*$^{+/-}$ (n=7), *Zbtb33*$^{-/y}$ (n=6) biological repeats.

(D-E) Quantification of absolute cell numbers of HSPC subpopulations (D) WT (n=8), *Zbtb33*$^{-/y}$ (n=8) biological repeats., and myeloid progenitor subtypes (E) WT (n=4), *Zbtb33*$^{-/y}$ (n=4) biological repeats., in the bone marrow of indicated mice.

(F-H) Percentages of indicated HSPC populations and mature cells in bone marrow from aged *Zbtb33*-knockout and wildtype mice. (F) WT (n=4), *Zbtb33*$^{-/y}$ (n=4) biological repeats.

*$P < 0.05$, ns: not significant.

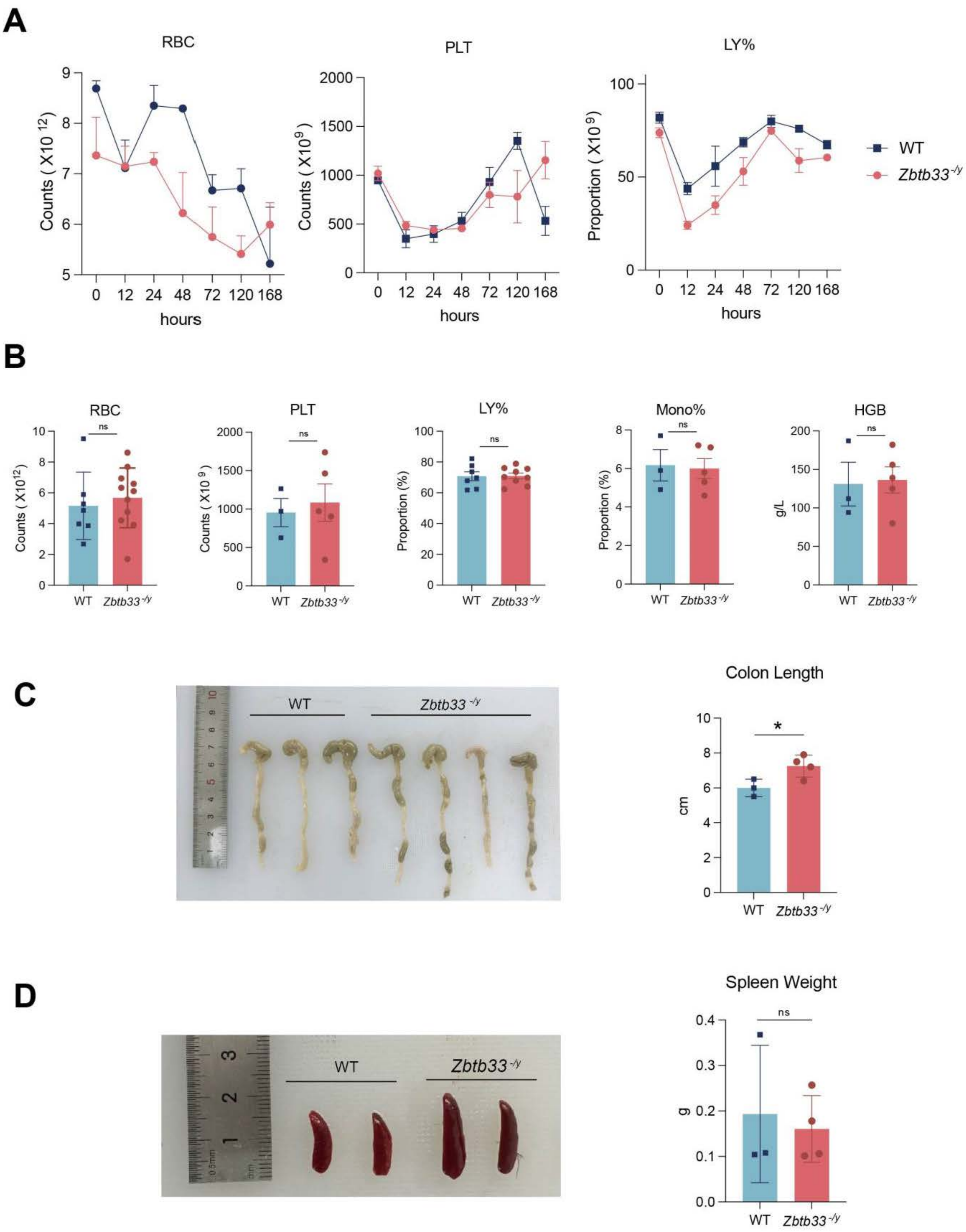

Disease Models & Mechanisms • Supplementary information

Fig. S2. related to Figure 4:

(A) Changes of peripheral blood cells at the indicated time point.

(B) Whole blood counts at 7 days post-DSS feeding in the context of loss of *Zbtb33* and wildtype genotype.

(C) Representative images and length measurement of integrate colons in DSS-administrated *Zbtb33*-knockout mice and the wildtype littermates.

(D) Photography and weight of spleens in DSS-induced inflammation models in the context of loss of *Zbtb33* and wildtype genotype.

*$P < 0.05$, ns: not significant. WT (n=3), *Zbtb33*$^{-/y}$ (n=4) biological repeats.

Disease Models & Mechanisms • Supplementary information

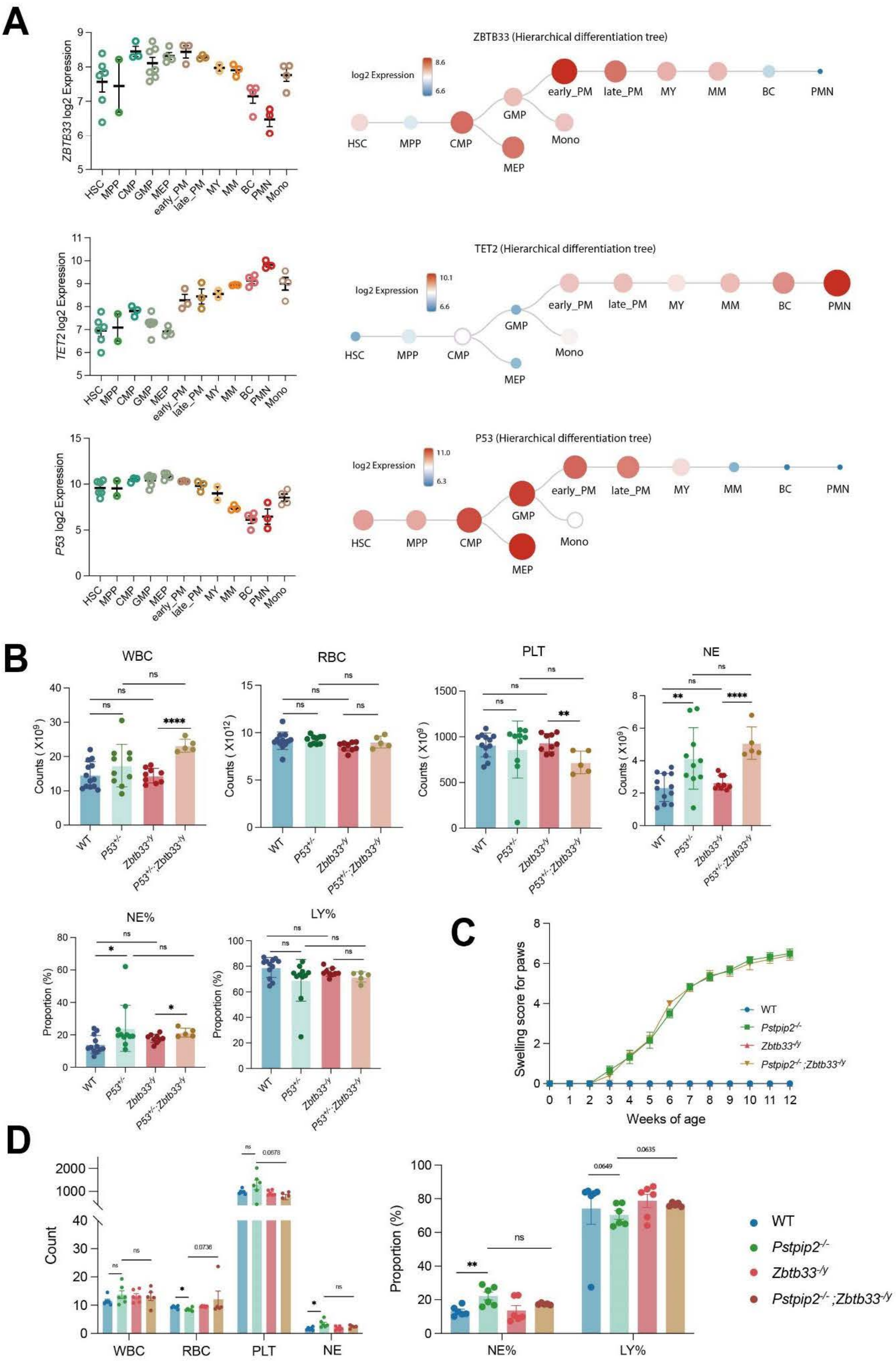

Disease Models & Mechanisms • Supplementary information

Fig. S3, related to Figure 5:

(A) Expression level of *ZBTB33*, *TET2*, and *P53* (mRNA, log_2) in indicated cells (source: the bloodspot database)

(B) Blood cell counts in WT, *P53*$^{+/-}$, *Zbtb33*$^{-/y}$, and *P53*$^{+/-}$*; Zbtb33*$^{-/y}$. WT (n=12), *P53*$^{+/-}$ (n=10), *Zbtb33*$^{-/y}$ (n=9), *P53*$^{+/-}$*; Zbtb33*$^{-/y}$ (n=5) biological repeats.

(**C-D**) Disease score quantification and PB cell counts in WT, *Pstpip2*$^{-/-}$, *Zbtb33*$^{-/y}$, and *Pstpip2*$^{-/-}$*; Zbtb33*$^{-/y}$. WT (n=6), *Pstpip2*$^{-/-}$ (n=6), *Zbtb33*$^{-/y}$ (n=6), *Pstpip2*$^{-/-}$*; Zbtb33*$^{-/y}$ (n=5) biological repeats.

P* < 0.05, ** *P* < 0.005, * *P* < 0.0005, **** *P* < 0.0001, ns: not significant.
